# Government Housing Assistance and Risk of Medical Financial Hardship Among Cancer Survivors

**DOI:** 10.1001/jamanetworkopen.2025.28976

**Published:** 2025-08-27

**Authors:** Katherine L. Chen, Carol M. Mangione, Ya-Chen Tina Shih

**Affiliations:** 1 Division of General Internal Medicine & Health Services Research, Department of Medicine, David Geffen School of Medicine, University of California, Los Angeles, Los Angeles; 2 Department of Health Policy and Management, Fielding School of Public Health, University of California, Los Angeles, Los Angeles; 3 Department of Radiation Oncology, David Geffen School of Medicine, University of California, Los Angeles, Los Angeles

## Abstract

This cross-sectional study examines the association between housing assistance and medical financial hardship among renters with a history of cancer.

## Introduction

Cancer is associated with elevated risk of medical financial hardship owing to out-of-pocket health care costs and disruptions to employment, income, and savings.^1^ High housing costs may contribute to this risk by limiting patients’ ability to absorb economic shocks.^2^ Meanwhile, cancer’s physical, cognitive, and financial toll may threaten vulnerable households’ housing stability.^1,2^ The interplay between housing costs and financial hardship is particularly concerning amid record-high rents burdens^3^ and increasing incidence of many cancers.^4^

Housing assistance (subsidized rent) increases low-income renters’ financial security^2^ and is associated with fewer cost-related health care barriers in the general population,^5^ but its association with cancer-related financial hardship is unknown. This study examined the association between housing assistance and medical financial hardship among renters with a history of cancer.

## Methods

This cross-sectional study followed the STROBE guidelines and was deemed exempt from review by the UCLA Jonsson Comprehensive Cancer Center. Using pooled National Health Interview Survey data (2019-2023), we included adult renters with a history of cancer, excluding those with nonmelanoma skin cancers and individuals missing values for study variables (5.2%). Respondents provided verbal consent. All variables were self-reported. Housing assistance was defined as paying subsidized rent. Medical financial hardship was defined as difficulty paying medical bills, missed or delayed care owing to costs, or worry about ability to pay unexpected medical bills (detailed definitions are shown in the eTable in Supplement 1).

We used weighted multivariable logistic models to regress medical financial hardship on housing assistance, controlling for sex, age, race and ethnicity, marital status, children in household, education, poverty level, employment, food assistance, supplemental security or disability payments, income assistance (termed *welfare* in the data source), health insurance, Medicare, Medicaid, self-rated health, chronic conditions, cancer site, time since diagnosis, region, and year. We report associations as differences in adjusted probabilities with 95% CIs to facilitate interpretation. Secondary analyses assessed associations with each financial hardship component and subgroup differences by age group and time since diagnosis. Data were analyzed using Stata MP statistical software version 17.0 (StataCorp). The threshold for statistical significance was set at 2-sided *P* < .05.

## Results

Among 2370 adult renters with cancer (1215 [39.7%] aged ≥ 65 years; 1568 [65.2%] female), 533 (19.7%) received housing assistance and 1270 (59.0%) reported medical financial hardship. Housing assistance recipients were disproportionately from minoritized racial and ethnic groups, had low income, were unemployed, and were in worse health (Table).

**Table.** Characteristics of Adult Renters With History of Cancer by Housing Assistance Receipt, National Health Interview Survey, 2019-2023

Characteristic	Participants, No. (%)^a^		
	Total (N = 2370 [100%])	Housing assistance (n = 533 [19.7%])	No housing assistance (n = 1837 [80.3%])
Age group, y			
18-39	261 (15.8)	27 (8.3)	234 (17.7)
40-64	894 (44.5)	195 (44.3)	699 (44.5)
≥65	1215 (39.7)	311 (47.4)	904 (37.8)
Gender			
Female	1568 (65.2)	376 (71.7)	1192 (63.6)
Male	802 (34.8)	157 (28.3)	645 (36.4)
Race and ethnicity^b^			
Asian, non-Hispanic	49 (3.1)	12 (3.2)	37 (3.0)
Black, non-Hispanic	337 (14.6)	120 (23.7)	217 (12.4)
Hispanic	287 (16.2)	77 (19.9)	210 (15.3)
White, non-Hispanic	1603 (61.9)	295 (48.1)	1308 (65.4)
Other or multiple, non-Hispanic^c^	94 (4.1)	29 (5.1)	65 (3.9)
Educational attainment			
Less than high school	335 (17.9)	128 (30.0)	207 (14.9)
High school graduate	602 (26.4)	153 (26.2)	449 (26.4)
Any associate degree or college	1433 (55.8)	252 (43.8)	1181 (58.7)
Marital status			
Married	536 (33.3)	71 (21.8)	465 (36.2)
Cohabiting	124 (8.7)	11 (3.8)	113 (9.9)
Not married or cohabiting	1710 (58.0)	451 (74.4)	1259 (54.0)
Minor children residing in household	310 (19.1)	61 (19.9)	249 (18.9)
Household income percentage of federal poverty level			
<99%	539 (22.6)	288 (51.5)	251 (15.5)
100%-199%	693 (30.6)	184 (37.6)	509 (28.9)
200%-399%	681 (27.7)	51 (9.3)	630 (32.2)
≥400%	457 (19.1)	10 (1.7)	447 (23.4)
Employed	840 (41.2)	62 (14.0)	778 (47.8)
Received food assistance in past year	708 (32.1)	331 (65.3)	377 (23.9)
Received Supplemental Security Income or Social Security Disability Income in past year	541 (25.4)	224 (45.2)	317 (20.5)
Received income assistance (welfare) or other public assistance in past year	178 (8.1)	84 (18.3)	94 (5.6)
Health insurance	2266 (93.0)	520 (97.2)	1746 (91.9)
Any Medicare	721 (24.0)	237 (37.9)	484 (20.6)
Any Medicaid	595 (26.8)	282 (56.3)	313 (19.6)
Self-rated health			
Poor or fair	968 (41.7)	315 (62.2)	653 (36.6)
Good, very good, or excellent	1402 (58.3)	218 (37.8)	1184 (63.4)
No. of chronic conditions^d^			
0	344 (17.1)	31 (6.0)	313 (19.9)
1-2	1048 (43.7)	190 (35.2)	858 (45.8)
3-4	776 (31.2)	231 (44.5)	545 (27.9)
≥5	202 (8.0)	81 (14.2)	121 (6.4)
Time since cancer diagnosis			
0-2 y	521 (22.2)	105 (20.4)	416 (22.7)
3-5 y	378 (17.4)	95 (18.1)	283 (17.3)
≥6 y	1471 (60.3)	333 (61.5)	1138 (60.1)
Cancer site			
Female breast	541 (19.6)	133 (23.2)	408 (18.8)
Colorectal	170 (7.2)	43 (10.4)	127 (6.4)
Prostate	237 (8.6)	45 (7.4)	192 (8.9)
Lung	124 (4.7)	38 (5.2)	86 (4.5)
All others	1298 (59.9)	274 (53.6)	1024 (61.4)
Medical financial hardship	1270 (59.0)	270 (53.7)	1000 (60.3)
Difficulty paying medical bills	482 (24.4)	95 (22.6)	387 (24.8)
Missed or delayed care due to cost	464 (21.9)	103 (21.5)	361 (21.9)
Worried about affording unexpected medical bills	1068 (49.6)	210 (42.5)	858 (51.4)
Region			
Northeast	421 (18.7)	130 (29.4)	291 (16.1)
Midwest	544 (20.9)	122 (19.6)	422 (21.3)
South	810 (36.3)	161 (30.5)	649 (37.7)
West	595 (24.1)	120 (20.6)	475 (24.9)
Survey year			
2019	588 (22.6)	139 (24.6)	449 (22.1)
2020	365 (21.0)	87 (20.8)	278 (21.0)
2021	482 (19.3)	96 (14.7)	386 (20.4)
2022	437 (18.3)	96 (19.2)	341 (18.1)
2023	498 (18.8)	115 (20.8)	383 (18.3)

^a^ Unweighted sample sizes and weighted frequencies are shown.

^b^ Included as proxy for structural racism in housing and health care.

^c^ Other included American Indian or Alaska Native, multiracial, or any other race and ethnicity not otherwise specified.

^d^ Number of chronic health conditions other than cancer was assessed as the sum of the number of affirmative responses to a personal history of arthritis; asthma or chronic obstructive pulmonary disease; diabetes; coronary heart disease, angina pectoris, or heart attack; hypertension; stroke; and anxiety or depression.

Participants with housing assistance exhibited a 6.7–percentage point lower adjusted probability of medical financial hardship vs those without assistance (Figure), including less difficulty paying medical bills and marginally less worry about unexpected bills, but no difference in missed or delayed care due to cost. Although stratified estimates varied, Wald tests indicated no interactions by age group or time since diagnosis (Figure).

**Figure.**
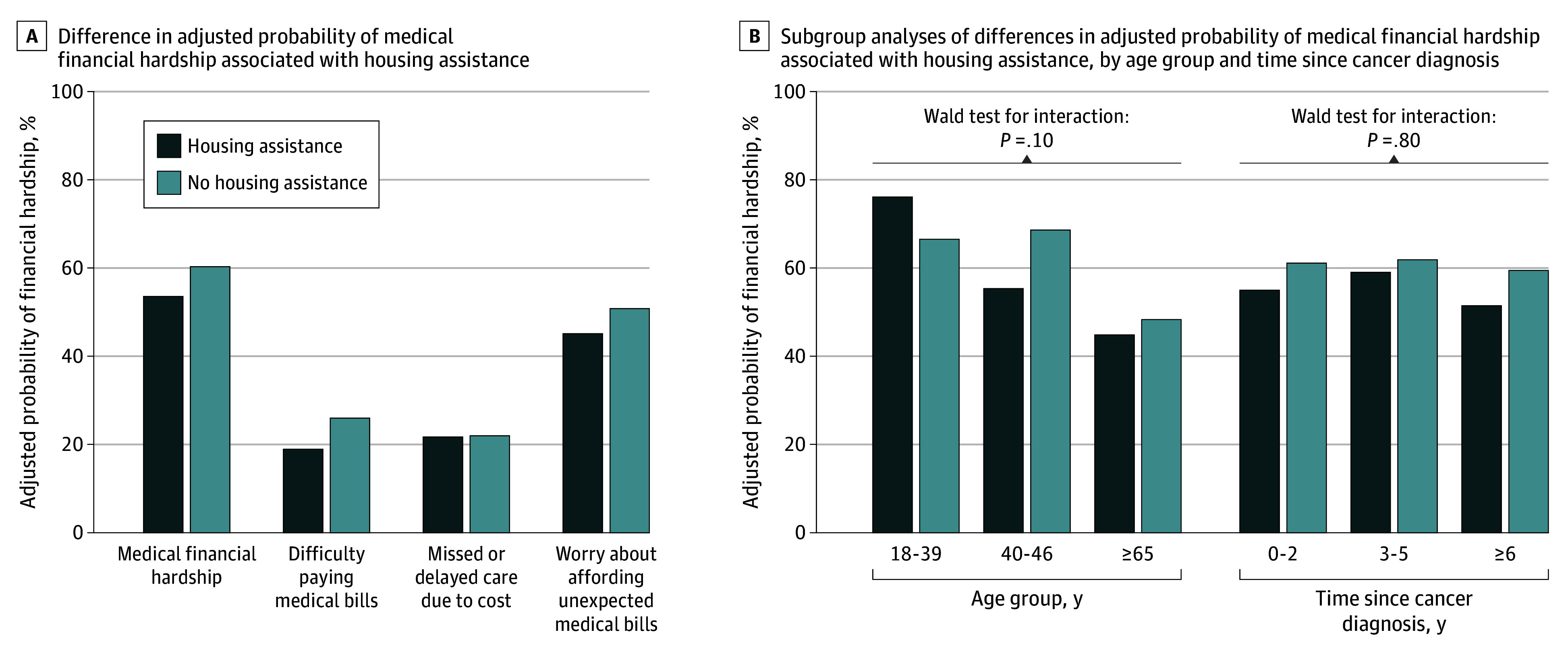
Associations Between Housing Assistance and Medical Financial Hardship Among Renters With a History of Cancer A, Adjusted probabilities of medical financial hardship and average marginal effects (differences in adjusted probabilities) associated with housing assistance vs no housing assistance were estimated from multivariable logistic regression models. For those with vs without housing assistance, the percentage point differences in adjusted probability were −6.7% (95% CI, −13.2% to −0.2%; *P* = .04) for medical financial hardship, −7.1% (95% CI, −12.6% to −1.6%; *P* = .01) for difficulty paying medical bills, −0.3% (95% CI, −5.5% to 5.0%; *P* = .92) for missed or delayed care due to cost, and −5.7% (95% CI, −12.4% to 1.0%; *P* = .10) for worry about affording unexpected medical bills. B, Subgroup differences by age group and time since cancer diagnosis were examined by separately adding interaction terms between housing assistance and each stratifying variable to the model for medical financial hardship; the overall significance of the interaction between housing assistance and each stratifying variable was determined using a Wald test of the joint significance of relevant interaction terms. For those with vs without housing assistance, percentage point differences in the adjusted probability of medical financial hardship were 9.6% (95% CI, −9.5% to 28.6%; *P* = .32) for those aged 18 to 39 years, −13.3% (95% CI, −23.4% to −3.3%; *P* = .009) for those aged 40 to 64 years, −3.5% (95% CI, −11.4% to 4.5%; *P* = .39) for those aged 65 years and older, −6.1% (95% CI, −20.5% to 8.3%; *P* = .40) for those 0 to 2 years since diagnosis, −2.9% (95% CI, −16.3% to 10.6%; *P* = .68) for those 3 to 5 years since diagnosis, and −8.0% (95% CI, −15.7% to −0.3%; *P* = .04) for those 6 or more years since diagnosis.

## Discussion

In this cross-sectional study, receipt of housing assistance was associated with lower risk of medical financial hardship among renters with a history of cancer. Those with assistance reported less difficulty with and worry about paying medical bills despite lower socioeconomic status and more health problems. This association was seen primarily among middle-aged adults, for whom challenges around careers and caregiving, combined with relatively high debt burden and limited safety-net support, may create unique financial vulnerabilities.^6^ The association with financial hardship risk persisted even 6 years after cancer diagnosis, suggesting an enduring benefit. However, subgroup-specific findings should be considered exploratory in the absence of significant interactions.

These results suggest that housing assistance may give renters a buffer to withstand cancer’s financial toll. Housing assistance may also facilitate better access to care, facilitating diagnosis at earlier, less costly stages.^2,5^ Given the known ties between cancer financial hardship, quality of life, and health,^1^ expanding housing assistance access could help mitigate financial hardship and improve well-being among cancer survivors. Since most eligible households do not receive housing assistance, this may require interventions to connect eligible patients to assistance (eg, via screening and referrals) and policy advocacy to increase, or at least maintain, government funding for assistance programs.^2^ Limitations include residual confounding (including unknown cancer stage and treatment history), survivorship bias, and uncertain housing assistance timing relative to financial hardship.

## Notes

Supplement 1. **eTable.** Definitions of Key Variables

Supplement 2.
Data Sharing Statement
