# Supplementary material for: Government Housing Assistance and Risk of Medical Financial Hardship Among Cancer Survivors
Source: JAMA Netw Open. 2025 Aug 27;8(8):e2528976. doi: 10.1001/jamanetworkopen.2025.28976 (PMC12391976; doi:10.1001/jamanetworkopen.2025.28976)
Supplement: Supplement 1. — eTable. Definitions of Key Variables [file jamanetwopen-e2528976-s001.pdf]

Supplemental Online Content

Chen KL, Mangione CM, Shih YT. Government housing assistance and risk of medical financial hardship among cancer survivors. *JAMA Netw Open*. 2025;8(8):e2528976.
doi:10.1001/jamanetworkopen.2025.28976

eTable. Definitions of Key Variables

This supplemental material has been provided by the authors to give readers additional information about their work.

© 2025 Chen KL et al. *JAMA Network Open*.

eTable. Definitions of Key Variables

Variable	Details
Renter status	Renters were identified as those who responded “rented” to the following question about their current residence: “Is the house/apartment…owned or being bought; rented; other arrangement; refused; don’t know.”
Housing assistance	Binary indicator for response to the question, “Are you paying lower rent because the Federal, State, or local government is paying part of the cost?” (Yes/No)<br><br>*Of note, the broad wording of this survey item precludes us from identifying specific eligibility criteria for housing assistance, which varies among assistance programs. In general, these programs use income-based thresholds to determine eligibility.*
Difficulty paying medical bills[a]	Binary indicator for response to the question, “In the past 12 months, did you/anyone in your family have problems paying or were unable to pay any medical bills?” (Yes/No)
Missed or delayed care due to costs[a]	Binary indicator for affirmative response to any of the following questions:<br>1. “During the past 12 months, have you DELAYED getting medical care because of the cost?”<br>2. “During the past 12 months, was there any time when you needed prescription medication, but DID NOT GET IT because of the cost?”<br>3. “During the past 12 months, was there any time when you needed medical care, but DID NOT GET IT because of the cost?”<br>4. “During the past 12 months, were any of the following true for you?<br>...You skipped medication doses to save money.<br>...You took less medication to save money.<br>...You DELAYED filling a prescription to save money.”<br>(Yes/No)
Worried about ability to pay unexpected medical bills[a]	Binary indicator for response to the question, “If you get sick or have an accident, how worried are you that you will be able to pay your medical bills? Are you very worried, somewhat worried, or not at all worried?” Responses of “worried” or “somewhat worried” were categorized as affirmative, and responses of “not at all worried” were categorized as negative. (Yes/No)
Medical financial hardship[a]	Summary variable, coded as binary indicator for presence of difficulty paying medical bills, missed or delayed care due to costs, OR worry about ability to pay unexpected medical bills (Yes/No)

[a]Coding for medical financial hardship variables in the National Health Interview Survey data was adapted from Yabroff KR, Doran JF, Zhao J, et al. Cancer diagnosis and treatment in working-age adults: Implications for employment, health insurance coverage, and financial hardship in the United States. *CA Cancer J Clin*. 2024;74(4):341-358. doi:10.3322/caac.2183.

© 2025 Chen KL et al. *JAMA Network Open*.
