# Supplementary material for: Government Housing Assistance and Risk of Medical Financial Hardship Among Cancer Survivors
Source: JAMA Netw Open. 2025 Aug 27;8(8):e2528976. doi: 10.1001/jamanetworkopen.2025.28976 (PMC12391976; doi:10.1001/jamanetworkopen.2025.28976)
Supplement: Supplement 2. — Data Sharing Statement [file jamanetwopen-e2528976-s002.pdf]

Data Sharing Statement

Chen. Government Housing Assistance and Risk of Medical Financial Hardship Among Cancer Survivors. *JAMA Netw Open*. Published August 27, 2025.
doi:10.1001/jamanetworkopen.2025.28976

Data
Data available: No

Additional Information
Explanation for why data not available: The study data are publicly available from the National Center for Health Statistics.
